# Supplementary material for: The Thousand Polish Genomes—A Database of Polish Variant Allele Frequencies
Source: Int J Mol Sci. 2022 Apr 20;23(9):4532. doi: 10.3390/ijms23094532 (PMC9104289; doi:10.3390/ijms23094532)
Supplement: Supplementary file 1 [file ijms-23-04532-s001.zip › ijms-1668894-supplementary.pdf]

<u>Supplementary information</u>

This Supplementary information includes:

Supplementary text – 5 notes
Figures S1 to S13
Tables S1 to S7
SI References

Other supplementary materials for this manuscript include the following:
Datasets – supplemental tables S6 and S7 (an Excel file)

Supplemental notes

Note S1: Runs of homozygosity

Our results show that on average, an individual genome contains 1.1 runs of homozygosity (RoH) longer than 100kb, spanning a total of 284Mb. RoH were uniformly distributed along the chromosomes, with the exception of centromeric and near-centromeric regions (Supplemental Figure S8). Mean size of a RoH was 252.7kb (100kb-14Mb), and counts and average sizes of RoH on particular chromosomes can be found in Supplemental Figure S10 and Supplementary Figure S11). The steepest growth in cumulative length of RoHs (Supplemental Figure S9) was observed between 20kb and 200kb, and RoH longer than 200kb represented less than a quarter of the homozygous sequence (median 88Mb).

Runs of homozygosity (ROH) analysis identifies the stretches of contiguous homozygous sites in an individual and are used to measure the level of inbreeding and recessive inheritance. The longer the total length of ROH in individuals the closer the inbreeding in a population, which increases the risk of recessive disease and decreases reproductive fitness of the offspring[1]. The Polish population characterised in this study matches the European ROH characteristics[2,3]. This suggests that the inbreeding level and potential risk of recessive diseases caused by homozygosity is relatively low.

There is however some risk that sampling bias could have influenced the ROH analysis as most of the volunteering participants were city inhabitants. On the contrary ROH density increases in rural areas, less mobile and more prone to close-kin marriages. Unfortunately, this type of bias occurs in most large scale genomic studies to date[4].

Note S2: Mitochondrial haplogroups

Using variant calls in the mitochondrial genome, we inferred haplogroups among 1,076 unrelated individuals. In 930 individuals with high quality haplogroup assignment the most abundant haplogroup was H with (43.5%) representatives, U with 192 (18.1%), J with 105 (9.9%), and T with 99 (9.3%) individuals (Supplemental Figure S12). The largest H sub-haplogroup was H1 (N=151; 28.5% of the H haplogroup), and a similar number of individuals was divided between subclades H2, H5, H6 and H11 (N=158; together 29.9% of the H haplogroup). The second most abundant sub-haplogroup in the cohort was U5 with 134 (25.4%) individuals.

The analysis of mtDNA haplogroups. clearly shows that for the majority of people in our cohort (43.5 %) the haplogroup H was assigned, which is consistent with previous results for Polish and Slavic populations[5–7]. Other studies on mitochondrial DNA show that Poles as a population are characterized by different European haplogroups, with dominance of West Eurasian, Central and Eastern European haplogroups[6–8]. It was also shown that the Polish population is almost indistinguishable from other European nations, except for the sub-haplogroups U4a and HV3a which are predominantly found in Poles and Russians [9]. In this project we confirmed the presence of the U4a haplogroup (50 individuals), which is assumed to be of central-european origin[8].

Note S3: Mendelian inconsistencies - *denovo* variation in family-based analysis

Using small variant genotypes in 97 parents-child trios we identified on average 351 *denovo* substitutions (284-507), and *denovo* 1,136 indels (977-1,411) per child. A total of 200 rare *denovo* SNVs affected the protein coding sequence (117 missense, 55 synonymous, 24 splice-site or region, and 4 stop-gain) corresponding to 0-4 exonic SNVs in 93 trios, and more than 4 (5-8) in four trios. On average, a child carried 2.06 exonic *denovo* SNVs.

Note S4: Overrepresented genetic variants in POL

SLC26A3

The results of our study confirm the high incidence of the p.Ile675_Arg676insIle variant in the ***SLC26A3*** gene in the Polish population. *SLC26A3* encodes for an intestinal Cl−/HCO3−, Na+-independent exchanger and is associated with congenital secretory chloride diarrhea (OMIM #214700). We identified p.Ile675_Arg676insIle variant in heterozygosity in 6 out of a total 7 pathogenic variants in *SLC26A3* in our cohort. In Poland variant p.Ile675_Arg676insIle is associated with 50% of all congenital chloride diarrhea cases. Similarly, a homogeneity of pathogenic variants in *SLC26A3* gene was reported in Finland, whereas in other European countries rare variants prevail[10].

MTMR2

For *MTMR2* gene we observed cumulative frequency of LoF SVs above 14.8% in POL. Homozygous and compound heterozygous variants in *MTMR2* are associated with Charcot-Marie-Tooth (CMT) disease type 4B1 - a rare autosomal recessive demyelinating neuropathy with usually severe course and early onset (OMIM # 601382). Although, to our best knowledge, no patients of Polish origin were

diagnosed with CMT 4B1, several laboratories have reported heterozygous, rare SVs among their patients (data unpublished).

Note S5: Selected disease causing variants - SLOS, NBS and CF

To demonstrate the utility of the database, we scrutinized variants distinctive for the Eastern European or the Slavic populations. The most frequent pathogenic mutation in POL was NG_012655.2:g.12031G>A, p.Trp151Ter variant (rs11555217) located in the *DHCR7* gene. It was carried by 26 unrelated participants (MAF=1.21%) indicating much higher frequency than observed in the GnomAD populations (Supplemental Figure S13 A; 0.07% in GnomAD v3.1.1). Mutations in *DHCR7* cause Smith-Lemli-Opitz syndrome (SLOS), an autosomal recessive disorder characterized by failure to thrive, microcephaly, intellectual disability, cleft palate and multiple birth defects, such as syndactyly of second and third toes, dysmorphic facial features or heart defects[11]. SLOS occurs as a common recessive disorder in Europe, with distinct mutations causing the disorder in different European populations. The founder p.Trp151Ter variant was the most frequent mutation among the Polish SLOS patients compared to patients from other subpopulations such as German or British[12]. The cumulative burden of pathogenic mutations in *DHCR7* in POL was almost 1.5 fold higher than in the NFE population (1.49% and 1.01%; Fisher p-value 0.038; Supplemental Table S6).

Another founder mutation, NM_002485.5:c.657_661del, resulting in p.Lys219fs variant (rs587776650) located in the *NBN* gene is responsible for the Nijmegen breakage syndrome (NBS). NBS is characterized by microcephaly, immunodeficiency and very high predisposition to lymphoid malignancy. It was reported that *NBN* heterozygosity is also responsible for increased incidence of tumors, especially lymphoma[13]. The disease occurs worldwide, but its prevalence was reported to be significantly higher in Poland, Ukraine, Czech Republic and Russia[14]. *NBN* (founder) mutation,

657del5, was estimated to appear in 1 case per 177 newborn[15], and has a global MAF in GnomAD 3.1.1 of 0.02% (GnomAD v3.1.1). In the POL cohort the frequency of this variant was almost seven fold higher than in the GnomAD NFE population (0.23% and 0.04% respectively; Fisher exact p-value 0.0027; Supplemental Figure S13 B).

Cystic Fibrosis (CF) is another example of an autosomal recessive disease, for which disease-causing allele spectrum was not derived from comprehensive genomic methods in the Polish population before. The analyses to date have utilized only targeted methods such as custom or commercial NGS panels or Sanger Sequencing of chosen exons[16,17]. Cystic fibrosis is the most frequent metabolic disease in Poland, with the incidence estimated at 1 per 4,394 [16] to 1 per 5,000 individuals [18]. CF affects mostly lungs and leads to progressive breathing problems and respiratory failure. The disease is caused by mutations located in *CFTR* gene (188 kb, 27 exons), for which more than 2,000 variants have been identified to date according to CFTR Mutation Database (see Web Resources). We observed that the cumulative frequency of Clinvar pathogenic variants in *CFTR* in the POL cohort and gnomAD NFE differ significantly (1.49% and 2.21%, respectively; Fisher p-value 0.024; Supplemental Table S6). Specifically, in the case of the most common NM_000492.3(CFTR):c.1521_1523delCTT (p.Phe508del, rs113993960), we observed that the allele frequency in POL is slightly lower (1.02%) than in NFE (1.43%), and much lower than previously reported 3% (NBS CF working group et al. 2013; Morral et al. 1994). Surprisingly, Slavic-specific NM_000492.3:c.54-5940_273+10250del (CFTRdele2,3) [20] variant was not found in the POL cohort in this study. It was reported before that, in the Polish population the p.Phe508del and CFTRdele2,3 mutations constituted 57-62% and 1.8-6.2% of all pathogenic CFTR mutations respectively (Bobadilla et al. 2002; NBS CF working group et al. 2013).

Supplemental figures

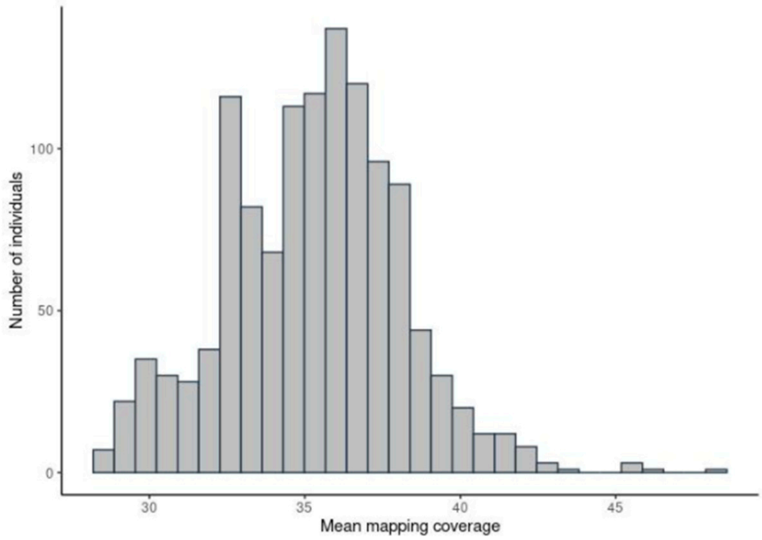

Figure S1: Distribution of mean mapping coverage in the analysed cohort.

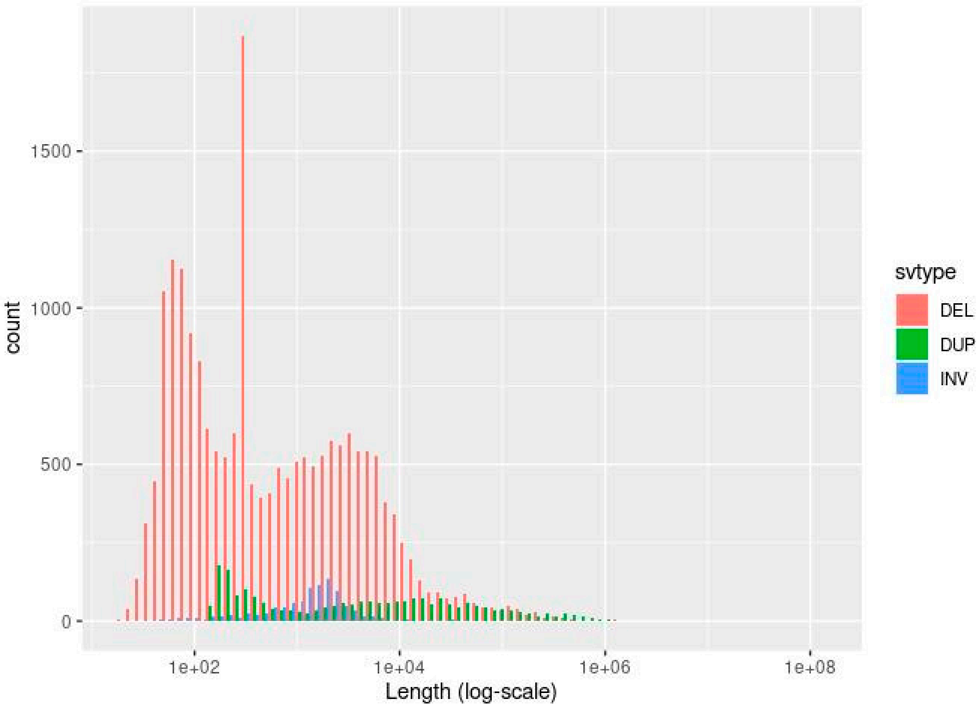

Figure S2 A: Distribution of structural variant (SV) lengths (log10 scale) for three SV types: deletions (DEL), duplications (DUP), and inversions (INV) among the analysed cohort.

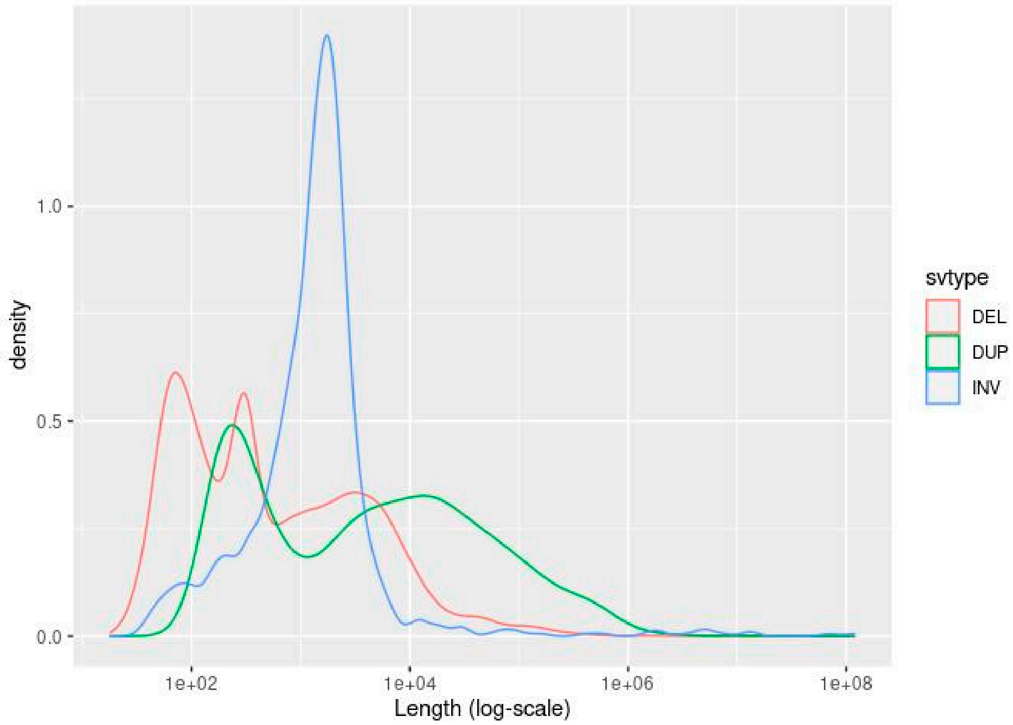

Figure S2 B: Distribution of structural variant (SV) lengths (log10 scale) presented as density for the three SV types: deletions (DEL), duplications (DUP), and inversions (INV) among the analysed cohort.

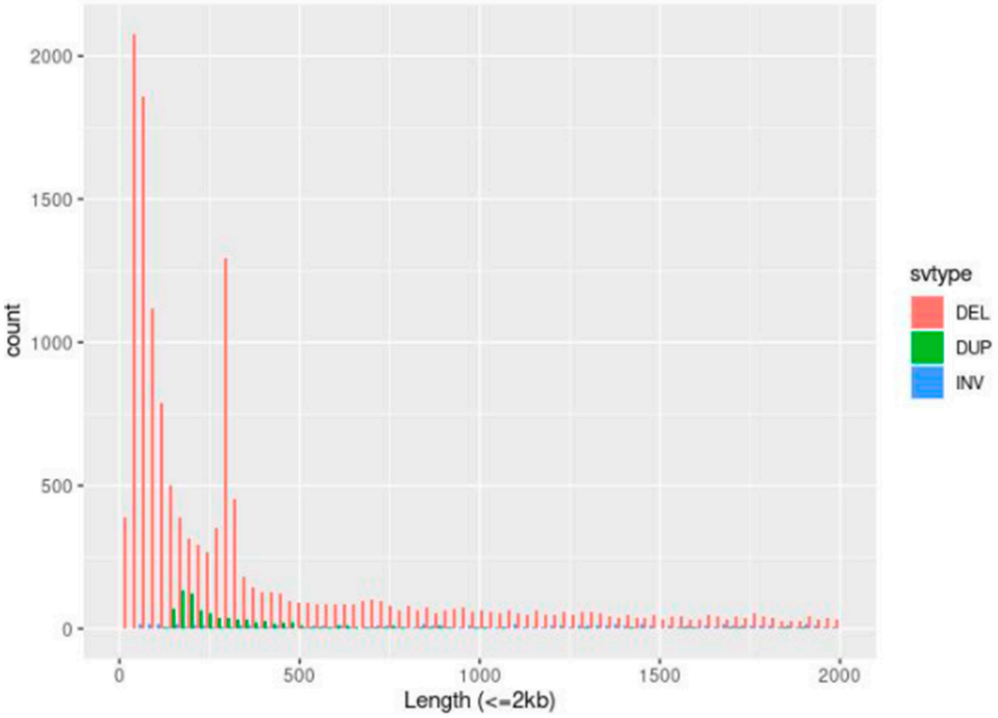

Figure S2 C: Distribution of structural variant (SV) lengths in the range 0-2000bp, for the three SV types: deletions (DEL), duplications (DUP), and inversions (INV) among the analysed cohort.

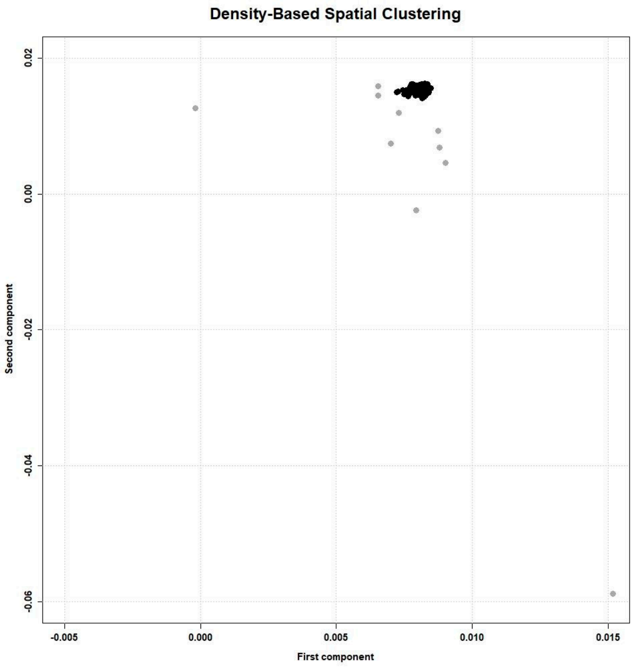

Figure S3: Density-based spatial clustering of the POL cohort shows homogeneity of the cohort with the exception of a few outliers. The average Fst statistics between the POL cluster and the outliers was 0.005, suggesting a non-significant difference between the individuals and the rest of the cohort.

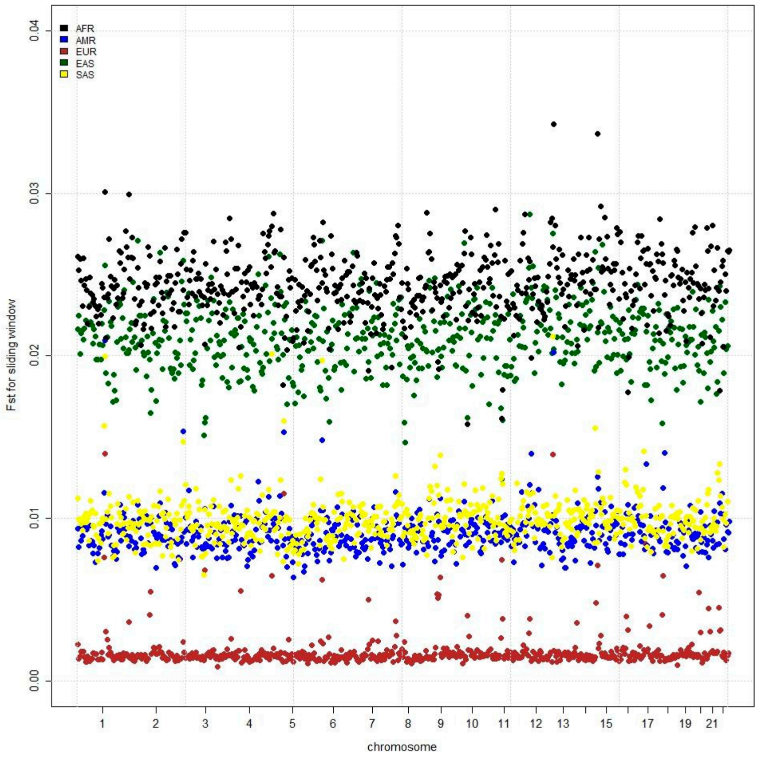

Figure S4: An average Fst statistics calculated over sliding windows of 1,000 SNPs for POL vs continental populations.

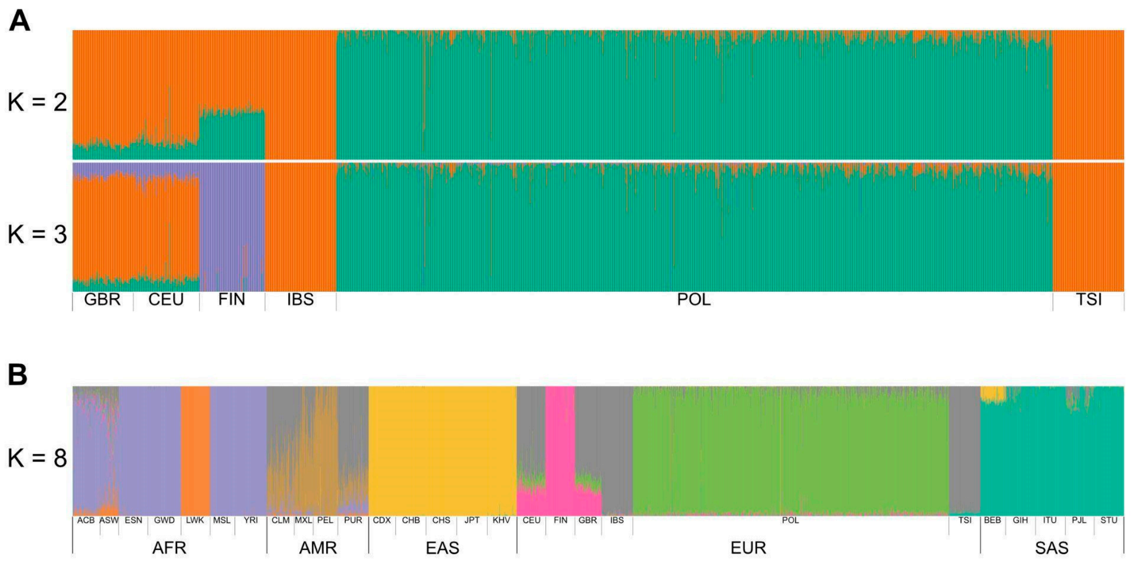

Figure S5: (A) Admixture[22] plots of the Polish cohort together with the European populations from the 1000 Genomes dataset. K = 3 minimizes the cross validation error value. Values of K = 2 and 3 were chosen based on the cross-validation error, and the MedMeaK, MaxMeaK, MedMedK and MaxMedK [23] estimators, respectively. **(B)** Admixture plot of the Polish cohort together with the entire 1000 Genomes world dataset. Value of K = 8 was chosen as in (A).

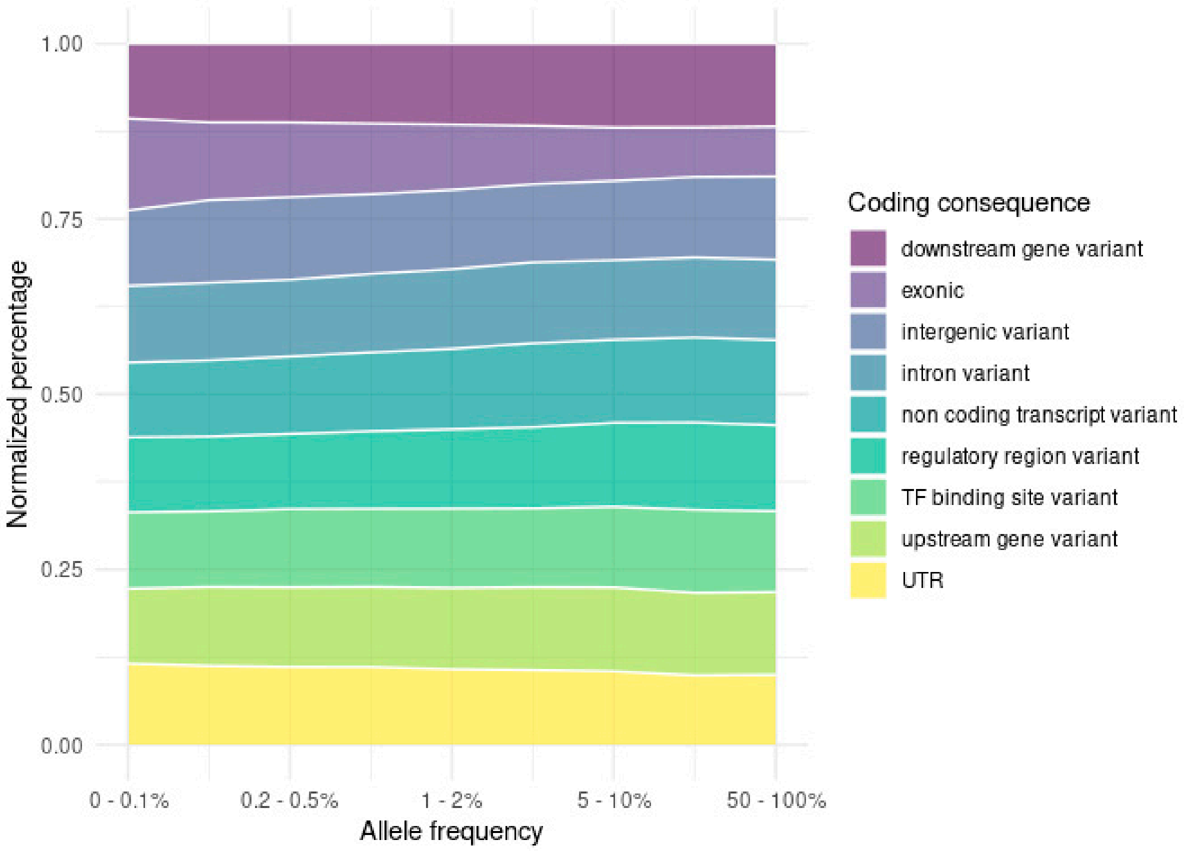

Figure S6: Distribution of variant consequences across allele frequency spectrum.

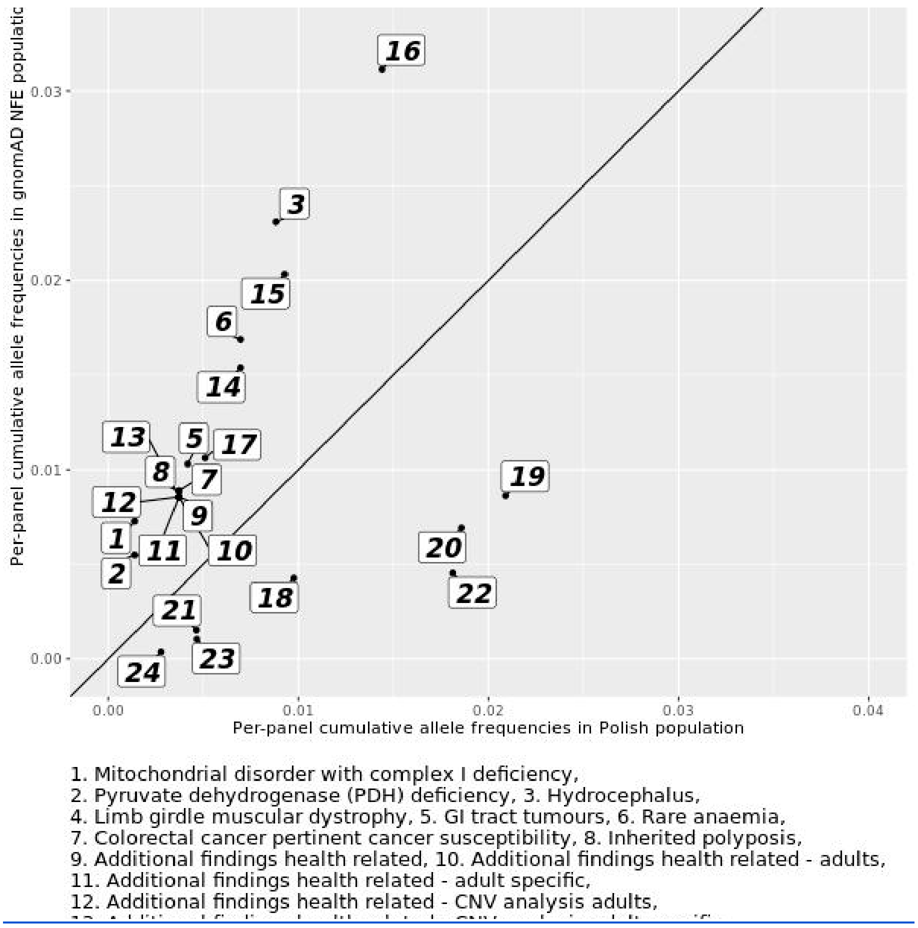

Figure S7: Cumulative allele frequencies of selected ClinVar variants in gene panels with significant differences between POL and GnomAD NFE populations.

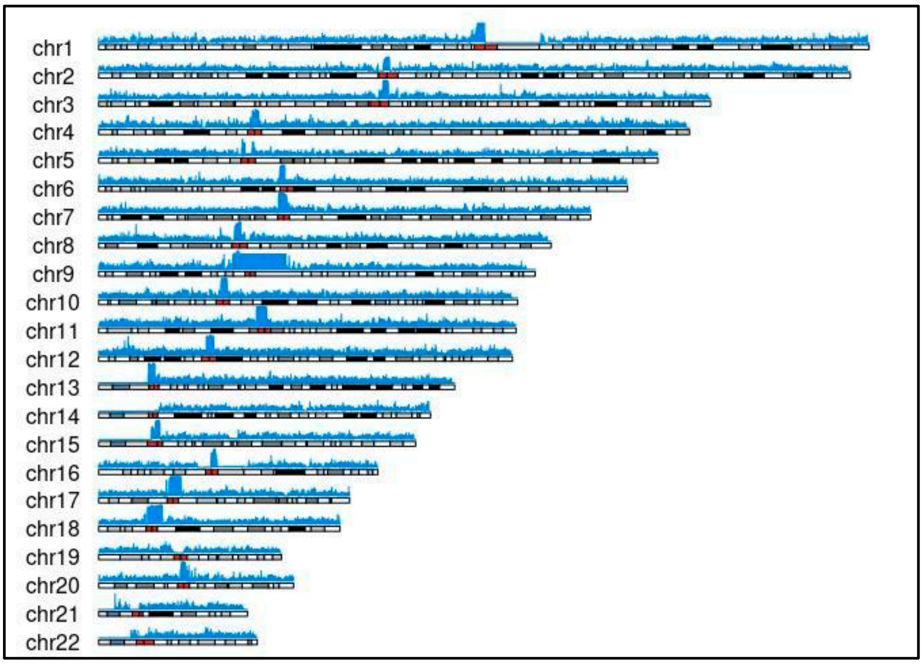

Figure S8: Chromosomes coverage by runs of homozygosity in the POL cohort.

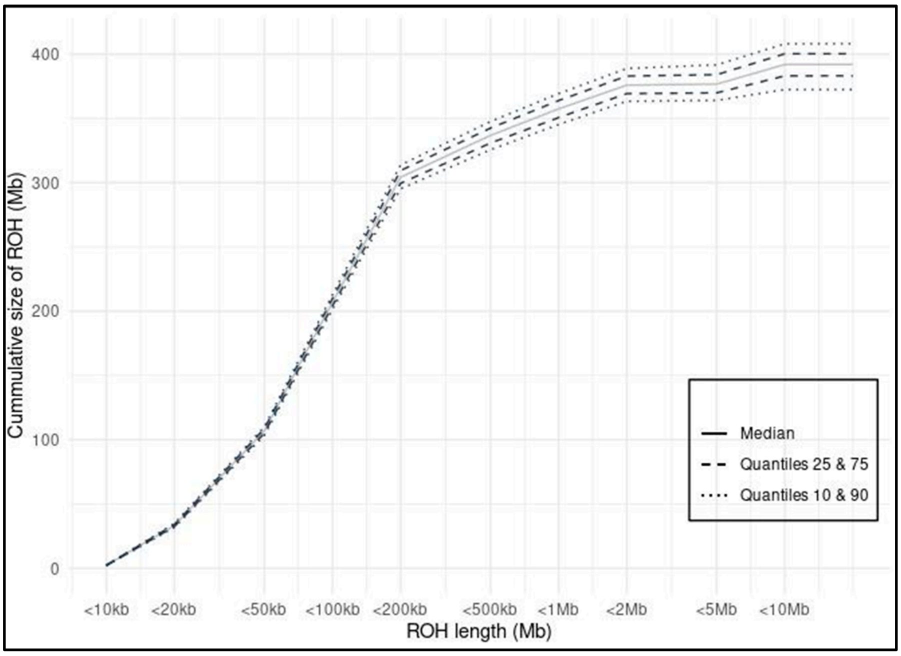

Figure S9: Cumulative length of RoHs.

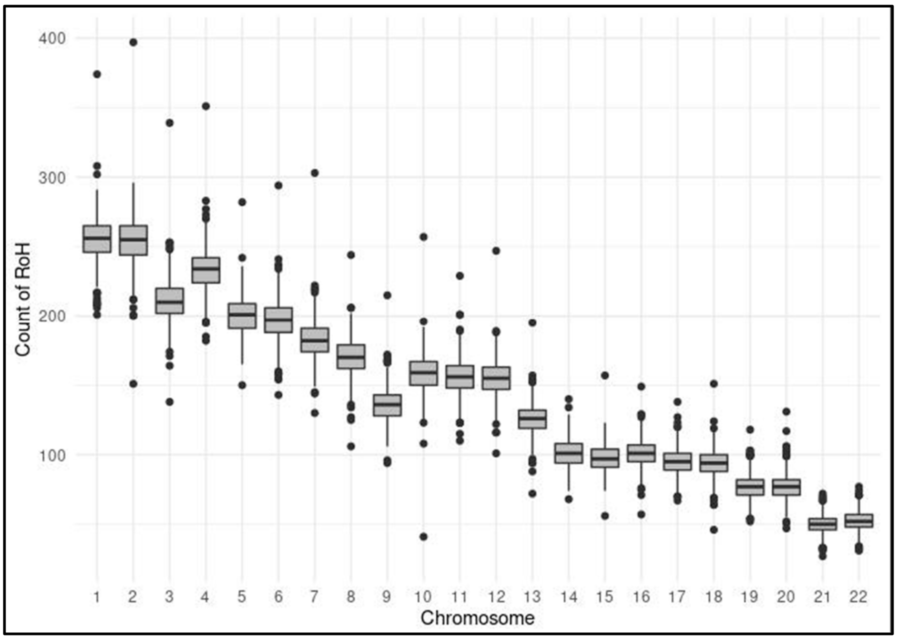

Figure S10: Number of Runs of homozygosity (RoHs) per chromosome. RoH analysis identifies the stretches of contiguous homozygous sites in an individual and are used to measure the level of inbreeding and recessive inheritance.

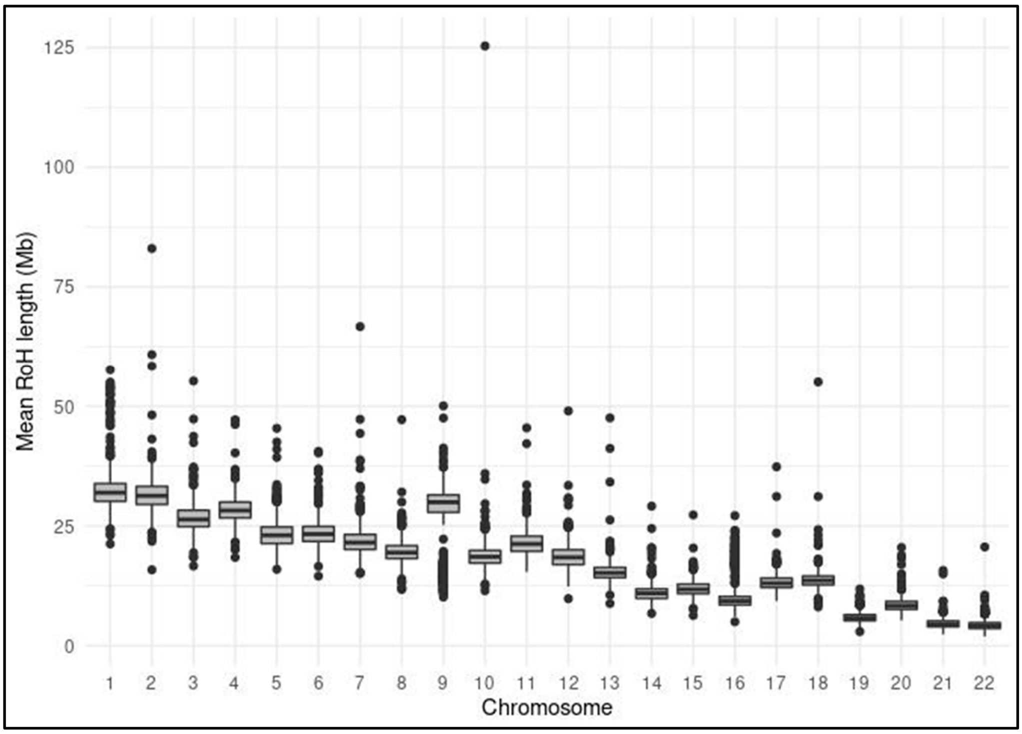

Figure S11: Average size of Runs of homozygosity (RoHs) per chromosome.

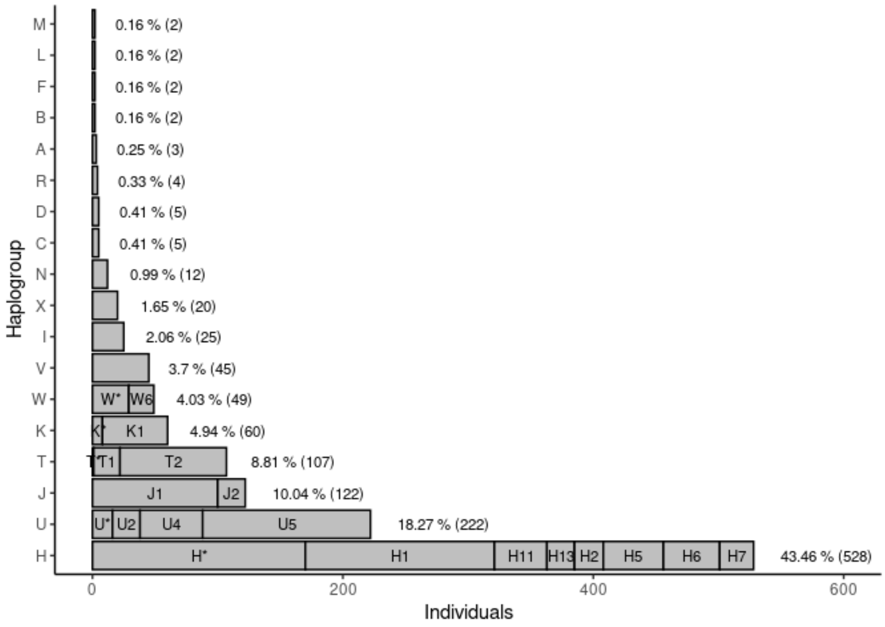

Figure S12: Haplogroups distribution in POL.

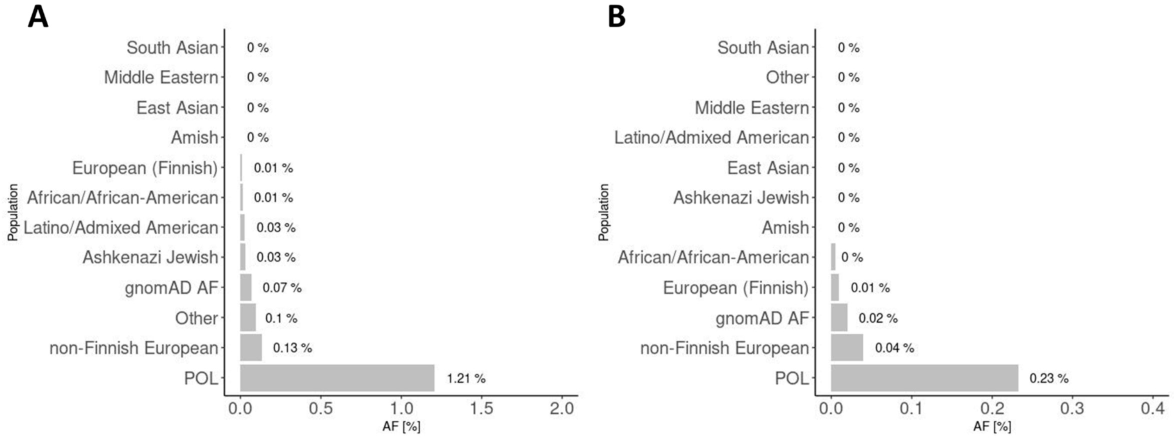

Figure S13: Frequency of selected variants in Polish (POL) and gnomAD v.3.1.1 populations: (A) the p.Trp151Ter variant (rs11555217) located in the *DHCR7* gene and (B).the 657del5 variant located in the *NBN* gene

Supplemental tables

Table S1: Coverage statistics for all 1,222 samples analysed in the project.

	Min	Median	Mean	Max
Average depth	28.40	35.41	35.26	48.10
Percent bp above 10X	91.40	91.93	91.90	92.41
Percent bp above 20X	84.15	89.11	89.19	91.27
Percent bp above 30X	49.05	78.69	76.84	90.00

Table S2: Small variant counts per individual. Singletons are SNVs, small insertions and deletions (indels) private to a single individual.

Variant	Min	Mean	Max
SNV	3,596,077	3,714,733	3,807,218
Indels	737,369	764,797	777,759
Singletons	3,202	15,877	123,142

Table S3: Total number (count), number of high quality (count.PASS) and percentage of high quality (pct.pass) structural variants in the dataset. BND-breakend, DEL-deletion, DUP-duplication, INV-inversion.

svtype	count	count.PASS	pct.pass
BND	21547	3964	0.1839699
DEL	45613	19808	0.4342622
DUP	14171	2270	0.1601863
INV	16800	998	0.0594048

Table S4: Numbers of structural variants per individual. BND-breakend, DEL-deletion, DUP-duplication, INV-inversion, het-heterozygous genotype, hom-homozygous genotype.

svtype	GT	min	mean	median	max
BND	het	156	216.50558	216.0	276
BND	hom	6	19.03717	18.0	34
DEL	het	1340	1514.50279	1512.5	1924
DEL	hom	671	1083.52881	1086.0	1200
DUP	het	56	74.54182	74.5	95
DUP	hom	21	31.76952	32.0	46
INV	het	46	64.65892	64.0	642
INV	hom	5	12.28717	12.0	21

Table S5: Fst statistics calculated for Polish cohort vs European subpopulations from the 1000 Genomes project vs world dataset.

Population	CEU	FIN	GBR	IBS	TSI
Fst	0.002	0.009	0.007	0.008	0.009

Supplemental materials and methods

Runs of homozygosity

Runs of homozygosity were identified using a hidden Markov model approach[24] implemented in BCFtools[25]. We analysed RoH Identified on autosomal chromosomes, requiring more than 50 markers per RoH, and average marker quality above 25.

Mitochondrial haplogroups

Small variants in the mitochondrial genome were called using Freebayes v.0.9.21[26], and mitochondrial haplogroups inferred using Haplogrep2 software[27].

Mendelian inconsistencies

Small variants violating Mendelian inheritance pattern were identified in 97 child-parents trios, and subsequently filtered using BCFtools[25]. For *de novo* candidates we selected sites covered by at least 10 reads in all family members, with minimum 5 alternative allele reads in the child and 0 such reads in the parents, and with minimum alt-allele depth ratio of 0.25. Additionally, only variants with Minor Allele Frequency (MAF)<1% in the cohort were kept.

Supplemental references

1. Hoffman, J.I., Simpson, F., David, P., Rijks, J.M., Kuiken, T., Thorne, M.A.S., Lacy, R.C., and Dasmahapatra, K.K. (2014). High-throughput sequencing reveals inbreeding depression in a natural population. Proc. Natl. Acad. Sci. *111*, 3775–3780.
2. Ceballos, F.C., Joshi, P.K., Clark, D.W., Ramsay, M., and Wilson, J.F. (2018). Runs of homozygosity: windows into population history and trait architecture. Nat. Rev. Genet. *19*, 220–234.
3. Pemberton, T.J., Absher, D., Feldman, M.W., Myers, R.M., Rosenberg, N.A., and Li, J.Z. (2012). Genomic Patterns of Homozygosity in Worldwide Human Populations. Am. J. Hum. Genet. *91*, 275–292.
4. Dean, C., Fogleman, A.J., Zahnd, W.E., Lipka, A.E., Malhi, R.S., Delfino, K.R., and Jenkins, W.D. (2017). Engaging rural communities in genetic research: challenges and opportunities. J. Community Genet. *8*, 209–219.
5. Grzybowski, T., Malyarchuk, B.A., Derenko, M.V., Perkova, M.A., Bednarek, J., and Woźniak, M. (2007). Complex interactions of the Eastern and Western Slavic populations with other European groups as revealed by mitochondrial DNA analysis. Forensic Sci. Int. Genet. *1*, 141–147.
6. Jarczak, J., Grochowalski, Ł., Marciniak, B., Lach, J., Słomka, M., Sobalska-Kwapis, M., Lorkiewicz, W., Pułaski, Ł., and Strapagiel, D. (2019). Mitochondrial DNA variability of the Polish population. Eur. J. Hum. Genet. *27*, 1304–1314.
7. Mielnik-Sikorska, M., Daca, P., Malyarchuk, B., Derenko, M., Skonieczna, K., Perkova, M., Dobosz, T., and Grzybowski, T. (2013). The History of Slavs Inferred from Complete Mitochondrial Genome Sequences. PLOS ONE *8*, e54360.
8. Malyarchuk, B.A., Grzybowski, T., Derenko, M.V., Czarny, J., Woźniak, M., and Miścicka-Sliwka, D. (2002). Mitochondrial DNA variability in Poles and Russians. Ann. Hum. Genet. *66*, 261–283.
9. Malyarchuk, B.A., Grzybowski, T., Derenko, M.V., Czarny, J., Woźniak, M., and Miścicka-Sliwka, D. (2002). Mitochondrial DNA variability in Poles and Russians. Ann. Hum. Genet. *66*, 261–283.
10. Wedenoja, S., Pekansaari, E., Höglund, P., Mäkelä, S., Holmberg, C., and Kere, J. (2011). Update on SLC26A3 mutations in congenital chloride diarrhea. Hum. Mutat. *32*, 715–722.
11. Ryan, A.K., Bartlett, K., Clayton, P., Eaton, S., Mills, L., Donnai, D., Winter, R.M., and Burn, J. (1998). Smith-Lemli-Opitz syndrome: a variable clinical and biochemical phenotype. J. Med. Genet. *35*, 558–565.
12. Witsch-Baumgartner, M., Ciara, E., Löffler, J., Menzel, H.J., Seedorf, U., Burn, J., Gillessen-Kaesbach, G., Hoffmann, G.F., Fitzky, B.U., Mundy, H., et al. (2001). Frequency gradients of DHCR7 mutations in patients with Smith-Lemli-Opitz syndrome in Europe: evidence for different origins of common mutations. Eur. J. Hum. Genet. *9*, 45–50.
13. Seemanová, E. (1990). An increased risk for malignant neoplasms in heterozygotes for a syndrome of microcephaly, normal intelligence, growth retardation, remarkable facies, immunodeficiency and chromosomal instability. Mutat. Res. *238*, 321–324.
14. Kostyuchenko, L., Makuch, H., Kitsera, N., Polishchuk, R., Markevych, N., and Akopian, H. (2009). Clinical immunology
Nijmegen breakage syndrome in Ukraine: diagnostics and follow-up. Cent. Eur. J. Immunol. *34*, 46–52.
15. Varon, R., Seemanova, E., Chrzanowska, K., Hnateyko, O., Piekutowska-Abramczuk, D., Krajewska-Walasek, M., Sykut-Cegielska, J., Sperling, K., and Reis, A. (2000). Clinical ascertainment

of Nijmegen breakage syndrome (NBS) and prevalence of the major mutation, 657del5, in three Slav populations. Eur. J. Hum. Genet. EJHG *8*, 900–902.
16. NBS CF working group, Sobczyńska-Tomaszewska, A., Ołtarzewski, M., Czerska, K., Wertheim-Tysarowska, K., Sands, D., Walkowiak, J., Bal, J., and Mazurczak, T. (2013). Newborn screening for cystic fibrosis: Polish 4 years' experience with CFTR sequencing strategy. Eur. J. Hum. Genet. *21*, 391–396.
17. Beauchamp, K.A., Johansen Taber, K.A., Grauman, P.V., Spurka, L., Lim-Harashima, J., Svenson, A., Goldberg, J.D., and Muzzey, D. (2019). Sequencing as a first-line methodology for cystic fibrosis carrier screening. Genet. Med. *21*, 2569–2576.
18. Farrell, P.M. (2008). The prevalence of cystic fibrosis in the European Union. J. Cyst. Fibros. *7*, 450–453.
19. Morral, N., Bertranpetit, J., Estivill, X., Nunes, V., Casals, T., Giménez, J., Reis, A., Varon-Mateeva, R., Macek, M., Kalaydjieva, L., et al. (1994). The origin of the major cystic fibrosis mutation (ΔF508) in European populations. Nat. Genet. *7*, 169–175.
20. Dörk, T., Macek, M., Mekus, F., Tümmler, B., Tzountzouris, J., Casals, T., Krebsová, A., Koudová, M., Sakmaryová, I., Macek, M., et al. (2000). Characterization of a novel 21-kb deletion, CFTRdele2,3(21 kb), in the CFTR gene: a cystic fibrosis mutation of Slavic origin common in Central and East Europe. Hum. Genet. *106*, 259–268.
21. Bobadilla, J.L., Macek, M., Fine, J.P., and Farrell, P.M. (2002). Cystic fibrosis: a worldwide analysis of CFTR mutations--correlation with incidence data and application to screening. Hum. Mutat. *19*, 575–606.
22. Alexander, D.H., Novembre, J., and Lange, K. (2009). Fast model-based estimation of ancestry in unrelated individuals. Genome Res. *19*, 1655–1664.
23. Puechmaille, S.J. (2016). The program structure does not reliably recover the correct population structure when sampling is uneven: subsampling and new estimators alleviate the problem. Mol. Ecol. Resour. *16*, 608–627.
24. Narasimhan, V., Danecek, P., Scally, A., Xue, Y., Tyler-Smith, C., and Durbin, R. (2016). BCFtools/RoH: a hidden Markov model approach for detecting autozygosity from next-generation sequencing data. Bioinformatics *32*, 1749–1751.
25. Danecek, P., Bonfield, J.K., Liddle, J., Marshall, J., Ohan, V., Pollard, M.O., Whitwham, A., Keane, T., McCarthy, S.A., Davies, R.M., et al. (2021). Twelve years of SAMtools and BCFtools. GigaScience *10*,.
26. Garrison, E., and Marth, G. (2012). Haplotype-based variant detection from short-read sequencing. ArXiv12073907 Q-Bio.
27. Weissensteiner, H., Pacher, D., Kloss-Brandstätter, A., Forer, L., Specht, G., Bandelt, H.-J., Kronenberg, F., Salas, A., and Schönherr, S. (2016). HaploGrep 2: mitochondrial haplogroup classification in the era of high-throughput sequencing. Nucleic Acids Res. *44*, W58-63.
